# DNA cytosine methylation in the Bovine Leukemia Virus promoter is associated with latency in a Lymphoma-derived B-cell line : potential involvement of direct inhibition of CREB/CREM/ATF binding

**DOI:** 10.1186/1742-4690-8-S1-A27

**Published:** 2011-06-06

**Authors:** Benoit Van Driessche, Laurence Colin, Allan Guiguen, Caroline Vanhulle, Jana Blazkova, Christelle Cardona, Makram Merimi, Michèle Nuttinck, Jean-Claude Twizere, Richard Kettmann, Daniel Portetelle, Arsène Burny, Ivan Hirsch, Olivier Rohr, Carine Van Lint

**Affiliations:** 1 Laboratoire de virologie moléculaire, Université Libre de Bruxelles, Gosselies, Belgium; 2 Institut de Cancérologie de Marseille, UMR599, Université de la Méditerranée, Marseille, France; 3 Laboratory of Experimental Hematology, Institut Jules Bordet, Université Libre de Bruxelles, Bruxelles, Belgium; 4 Département de Biologie Moléculaire, Faculté Universitaire des Sciences Agronomiques de Gembloux, Gembloux, Belgium; 5 Institut Universitaire de Technologie Louis Pasteur de Schiltigheim, Université de Strasbourg, Schiltigheim, France

## 

Bovine leukemia virus (BLV) proviral latency represents a viral strategy to escape the host immune system and allow tumor development. Besides the previously demonstrated role of histone deacetylation in the epigenetic repression of BLV expression, we showed here that BLV promoter activity was induced by several DNA methylation inhibitors (such as 5-aza-2'-deoxycytidine) and that overexpressed DNMT1 and DNMT3A, but not DNMT3B, down-regulated BLV promoter activity. Importantly, cytosine hypermethylation in the 5'-long terminal repeat (LTR) U3 and R regions was associated with true latency in the lymphoma-derived B-cell line L267 but not with defective latency in YR2 cells. Moreover, the virus-encoded transactivator Tax(BLV) decreased DNA methyltransferase expression levels, which could explain the lower level of cytosine methylation observed in the L267(LTaxSN) 5'-LTR compared with the L267 5'-LTR. Interestingly, DNA methylation inhibitors and Tax(BLV) synergistically activated BLV promoter transcriptional activity in a cAMP-responsive element (CRE)-dependent manner. Mechanistically, methylation at the -154 or -129 CpG position (relative to the transcription start site) impaired in vitro binding of CRE-binding protein (CREB) transcription factors to their respective CRE sites. Methylation at -129 CpG alone was sufficient to decrease BLV promoter-driven reporter gene expression by 2-fold. We demonstrated in vivo the recruitment of CREB/CRE modulator (CREM) and to a lesser extent activating transcription factor-1 (ATF-1) to the hypomethylated CRE region of the YR2 5'-LTR, whereas we detected no CREB/CREM/ATF recruitment to the hypermethylated corresponding region in the L267 cells. Altogether, these findings suggest that site-specific DNA methylation of the BLV promoter represses viral transcription by directly inhibiting transcription factor binding, thereby contributing to true proviral latency.

